# Loss-Modulation-Based Wavelength-Range Shifting of Tunable EDF Ring Laser with Cascaded-Chirped Long-Period Fiber Grating for Temperature Measurement †

**DOI:** 10.3390/s21072342

**Published:** 2021-03-27

**Authors:** Koken Fukushima, Manuel Guterres Soares, Atsushi Wada, Satoshi Tanaka, Fumihiko Ito

**Affiliations:** 1 Department of Communications Engineering, National Defense Academy, 1-10-20 Hashirimizu, Kanagawa, Yokosuka 239-8686, Japan; em58049@nda.ac.jp (M.G.S.); a24wada@nda.ac.jp (A.W.); 2 Graduate School of Natural Science and Technology, Shimane University, 1060 Nishikawatsu-cho, Shimane, Matsue 690-8504, Japan; ito@ecs.shimane-u.ac.jp

**Keywords:** tunable lasers, optical fiber sensors, long-period fiber gratings (LPG), cascaded-chirped long-period fiber gratings (C-CLPG), Erbium-doped fiber ring lasers (EDFRL), variable optical attenuator (VOA), intracavity loss modulation, temperature sensing

## Abstract

A novel tunable Erbium-doped fiber ring laser (EDFRL) with a cascaded-chirped long-period fiber grating (C-CLPG) as a wavelength selection filter is proposed from the viewpoint of the sensor use, in which a variable optical attenuator (VOA) is employed as an intracavity loss modulator to change the oscillation wavelength region so that the resultant tuning wavelength range is widened. In the demonstrative experiment for temperature measurements, oscillation over the wavelength range of 12.85 nm (1557.62~1570.47 nm), which is more than three times range of the previously presented laser and is equivalent to 64 °C in terms of temperature change, was achieved, while a single-wavelength oscillation was maintained. In addition, a practical technique for realizing a temperature measurement by combining with the VOA control is also discussed.

## 1. Introduction

Among the optical fiber sensors, fiber grating sensors have attracted much attention because they have a lot of advantages for practical sensor use: capability of localized and/or multiplexed sensing, high consistency with transmission fibers, ability to be embedded in structures, etc. In general, fiber gratings are fabricated by inducing a periodic refractive index modulation along the optical fiber, and are classified into two types based on their grating period: fiber Bragg grating (FBG) and long-period fiber grating (LPG). The FBG, which is inscribed typically with a submicron grating period, couples a forward-propagating core mode with a backward-propagating one at the Bragg wavelength, and serves as a narrow-band reflection filter [1]. In contrast, the LPG, which is inscribed with a grating period ranging from tens to hundreds of µm, couples a forward-propagating core mode with several codirectional cladding modes at their resonant wavelengths and provides a relatively wide and nonreflective band-rejection filter [2]. Especially for sensor applications, LPGs have advantages such as easier and more flexible in fabrication and design than FBGs due to their large grating period. In addition, LPG sensors utilize coupling characteristics between the core mode and the cladding modes, which makes them sensitive to strain, pressure, temperature, and the environmental refractive index [3].

In recent years, we have proposed several fiber-optic sensors in which a pair of LPGs or chirped LPGs (CLPGs) provides an in-fiber Mach–Zehnder interferometer, called a cascaded LPG (C-LPG) or a cascaded CLPG (C-CLPG), as the sensor element. In such sensors, periodic interference fringes (namely channeled spectra) appear around the attenuation dips of LPGs or CLPGs in a wavelength domain, and to achieve a highly sensitive and accurate sensing, an interference-fringe measurement method based on a Fourier transform technique focusing on the periodic channeled spectrum has been adopted. However, this method requires time-consuming data processing, which limits the measurand to static or quasistatic quantities [4,5]. To solve the above problems, we constructed an Erbium-doped fiber ring laser (EDFRL) with a C-CLPG as a wavelength selection filter and an Erbium-doped fiber amplifier (EDFA) as a gain medium, and have previously presented a method of direct sensing from the oscillation wavelengths [6]. As a result of the demonstrative experiment for the temperature measurement, the oscillation output was obtained at the peak wavelength in the transmittance channeled spectrum of the C-CLPG, and it exhibited a linear response to the temperature variation in accordance with that of the channeled spectrum. The oscillation wavelength range, however, was limited within a fringe spacing of the periodic channeled spectrum so that periodic sawtooth wavelength shifts were observed with the large temperature variations. Although a simple numerical compensation was also proposed to solve the limitation, it was required to know an accurate fringe spacing of the channeled spectrum and the corresponding temperature variation range a priori. Therefore, it was necessary to provide a mechanism to widen or shift the oscillation wavelength range of the laser during the tuning operation [7,8]. The purpose of this study is to extend the range of single-wavelength oscillation by a simple method to solve the problem of the limitation of the oscillation wavelength range mentioned above. In addition, such a laser operation is also very useful in light-source applications as a tunable and switching laser [9,10].

Several simple methods have been reported to extend or shift the oscillation wavelength of fiber ring lasers by adjusting the additional loss in the ring cavity. For the tunable filterless EDFRLs, P. Franco et al. [11] reported tuning the laser by adjusting a variable optical attenuator (VOA) inserted in the cavity to shift the net gain peak, and M. Melo et al. [12] reported that even a bending loss of a tapered single-mode fiber can be effectively used for the tuning operation of the laser. D. Venkatesh et al. [13] proposed a wavelength tuning method by controlling intracavity losses using a VOA as well as a variable optical coupler (VOC), and showed that a more stable output power with the less tuning-induced output variations was obtained by using VOA rather than VOC. For EDFRLs using comblike wavelength selection filters, Y. Liu et al. [14] reported a wavelength-switchable EDFRL using a sampled FBG and a VOA, and F. Li et al. [15] reported that the wavelength range of multiwavelength oscillations can be shifted by tuning the VOA losses. Recently, A. D. Guzman-Chavez et al. [16] demonstrated a wavelength-range tunable and switchable multiline fiber ring laser by using a VOA.

In this paper, a programmable VOA was employed as an intracavity loss modulator in the previously proposed EDFRL [6], in which a C-CLPG with a periodic channeled spectrum was used as a wavelength selection filter. In a wavelength-tunable laser using a comblike periodic wavelength filter such as the C-CLPG, the wavelength tunable range is generally limited within the period of the wavelength selective element, which limits the measurement range of the sensor using the oscillating wavelength, but by adjusting the loss and changing the oscillating wavelength range, the wavelength tunable range, or measurement range, can be expanded. We reported at the OECC 2020 international conference [17] that a wavelength-switching operation of the EDF ring laser using C-CLPG can be realized by varying the intracavity loss using a VOA. In this paper, however, we have conducted new experiments, and investigated the intracavity loss dependent characteristics of the laser to clarify the effect on the oscillation wavelength, and demonstrated a detailed examination of the tuning of the tunable wavelength range by loss from the viewpoint of improvement of the sensor applications. By applying a simple method to adjust the additional loss of the VOA used, it was possible to expand the effective tuning range of the oscillation wavelength and extend the measurement range in temperature sensing. In the experiment, we investigated the oscillation wavelength with temperature variation around the C-CLPG by changing the additional loss of VOA and examined the wavelength tuning range. As a result, we succeeded in expanding the maximum oscillation wavelength range to 1557.62~1570.47 nm (12.85 nm), which corresponds to a temperature-measurement range of ~64 °C, three times larger than that of the previous laser.

## 2. Apparatus

**Figure 1:**
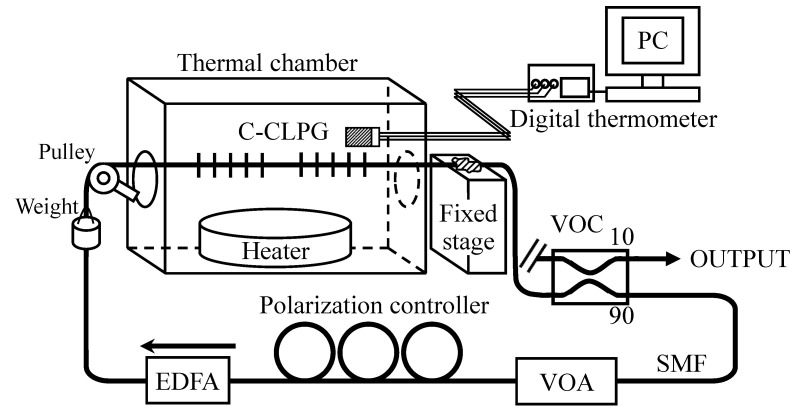
Experimental setup for the tunable and switchable EDFRL using C-CLPG.

The experimental setup is shown in Figure 1. The EDFRL consists of a C-CLPG as a sensing head, an EDFA (Mitsubishi Cable, FA155D-168FS) that includes built-in input and output isolators, a programmable VOA (Anritsu, MN9611B) to control the intracavity loss, and a VOC (KS Photonics, FTDC-N-155-1-FA) with a splitting ratio of 10:90. The oscillation spectrum of the output from the one port of the VOC is measured by means of an optical spectrum analyzer (OSA; Anritsu, MS9740A) with a resolution of 0.03 nm, and the split light from the other port is fed back into the EDFA through the VOA and a polarization controller. The C-CLPG was fabricated in such a way that the pair of CLPGs was written into a photosensitive fiber (Nufern, GF1AA) by a point-by-point method using a focused KrF excimer laser (GAM laser, EX10) beam (grating period: 300~305 µm; separation between two CLPGs: 100 mm; number of the period of the grating: 20; CLPG length: 5740 µm; chirp ratio: 0.871 × 10^−3^; and the full-width at half-maximum (FWHM) of the attenuation dip of the CLPG around 1560 nm: ~70 nm). The resultant channeled spectrum was obtained around 1560 nm in the transmittance spectrum of the C-CLPG. The EDF was backward-pumped by a 1.48 µm laser diode driven with an injection current of 100 mA, so that the oscillation was obtained at the peaks of the channeled spectrum around 1560 nm that corresponded to the maximum gain [17].

In this configuration the VOA enables the control of the amount of optical feedback to the EDFA by changing the additional intracavity losses. As the intracavity loss is reduced and the intensity of the feedback light to the EDFA is increased, the gain of the EDFA becomes deeply saturated. As a result, the gain profile is flattened over a wide wavelength range, and the maximum gain is obtained at a longer wavelength side [18], so that the oscillation can also be obtained at a longer wavelength side. On the other hand, as the intracavity loss is increased, the gain profile becomes similarly to the ASE curve, which is close to small signal gain, resulting in an oscillation obtained at a shorter wavelength side [11,19]. Therefore, the oscillation wavelength range of the EDF laser can be effectively controlled to the longer or shorter wavelength side by controlling the additional loss of the VOA.

## 3. Experimental Results and Discussions

### 3.1. Wavelength-Switching Operation

**Figure 2:**
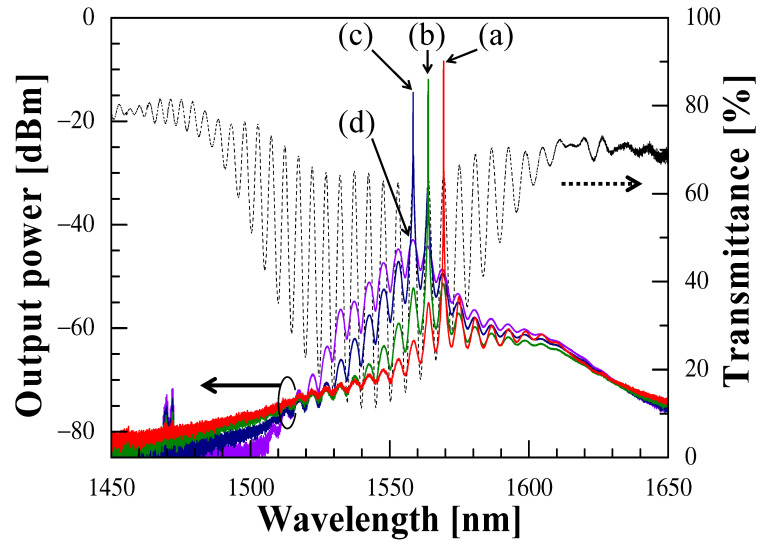
Oscillation spectra of the EDFRL at room temperature (~29 °C) with different VOA losses: (**a**) 0 dB, (**b**) 7 dB, (**c**) 15 dB, and (**d**) 30 dB.

Figure 2 shows the typical oscillation spectra for different intracavity losses (colored solid lines) and C-CLPG transmittance spectrum (black dotted line) at room temperature (~29 °C). It is noted that the oscillation spectrum, as well as the channeled spectrum of the C-CLPG remains constant without shifting if there is no variation in temperature. Since the oscillation output is usually obtained at one of the peaks of the channeled spectrum, as can be seen from the figure, the outputs of −8.39 dBm, −11.91 dBm, and −14.47 dBm were respectively obtained at 1569.35 nm (Ch.3), 1563.87 nm (Ch.2), and 1558.44 nm (Ch.1), when the respective VOA loss was set to 0, 7, and 15 dB. The reason for the wavelength-switching behaviors to the shorter wavelength for the higher VOA loss was that the oscillation wavelength range shifted to the shorter wavelength side as the intracavity loss of the VOA increased, as described in the previous section. Furthermore, when the intracavity loss increased more than 30 dB, no oscillation occurred, and only the output corresponding to the ASE light transmitted through the C-CLPG was observed.

### 3.2. Output Characteristics Dependent on Intracavity Loss

**Figure 3:**
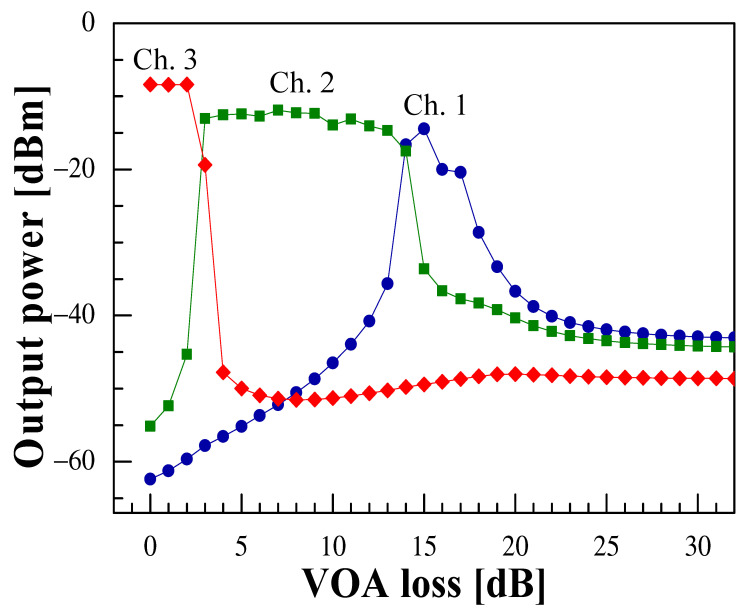
VOA loss-dependent output power at peak wavelengths of channeled spectra for Ch. 1 (blue circle), Ch. 2 (green square), and Ch. 3 (red diamond).

The oscillation output behavior was investigated in more detail with the intracavity additional loss of VOA varied by 1 dB. The output power obtained at each channel as a function of the VOA loss is shown in Figure 3. As shown in the figure, if the loss of VOA was set to 0 dB, oscillation occurred in Ch. 3. When the intracavity loss was gradually increased, the oscillation output in Ch. 3 decreased and stopped, while oscillation occurred in Ch. 2. As the intracavity loss was further increased, oscillation was obtained in Ch. 1 as well. These results reflect the fact that the oscillation wavelength range shifted to the short wavelength side as the VOA loss increased. In addition, since the EDFA was likely to be a homogeneous broadening gain media, single wavelength oscillation tended to occur essentially. However, as shown in the figure, when the loss of the VOA was about 3 dB and 14 dB, simultaneous dual-wavelength oscillations were obtained in the adjacent channels Ch. 3 and Ch. 1, and Ch. 2 and Ch. 1, respectively. These results indicated that the laser provided not only the wavelength-switching operation according to a peak of the channeled spectrum of the C-CLPG by tuning the VOA under the constant temperature, but it also was possible to switch between the single- and dual-wavelength operation, providing a laser with high potential as a light source.

### 3.3. Temperature Characteristics

**Figure 4:**
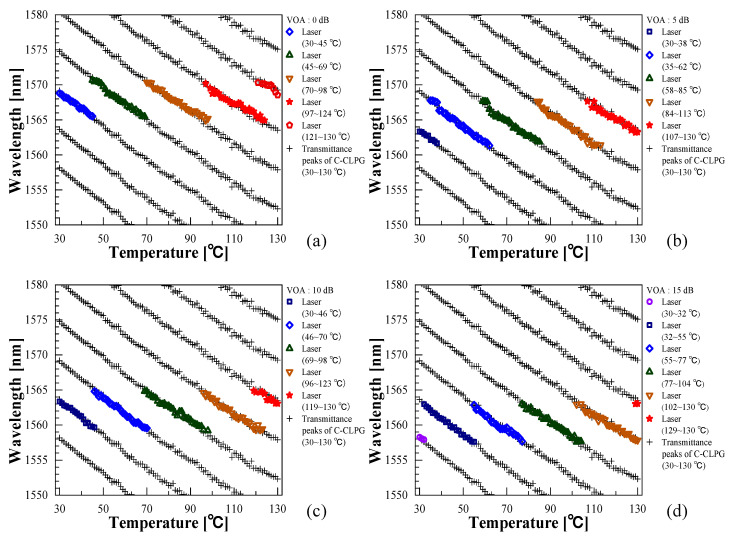
Oscillation wavelengths of EDFRL (colored symbol plots) and transmittance peak wavelengths of the C-CLPG (black cross plots) as a function of temperature for different VOA losses: (**a**) 0 dB, (**b**) 5 dB, (**c**) 10 dB, and (**d**) 15 dB.

Figure 4a–d show the temperature dependence of the EDFRL oscillation wavelengths extracted with the threshold at −30 dBm for different intracavity losses (colored symbol plots) and the peak wavelengths of the channeled spectrum of the C-CLPG (black cross plots). It was confirmed that the additional loss in the cavity allowed switching the oscillation wavelength range toward the shorter wavelength side. As can be seen from the figure, the oscillation wavelength ranges were obtained as follows, 1564.94~1570.68 nm (5.74 nm), 1561.34~1567.76 nm (6.42 nm), 1559.18~1564.89 nm (5.71 nm), and 1557.57~1563.05 nm (5.48 nm), respectively, for the VOA losses of 0 dB (a), 5 dB (b), 10 dB (c), and 15 dB (d). In this study, the oscillation wavelength corresponded to the temperature dependence of the channeled spectrum of the C-CLPG, which showed a linear response to temperature with a sensitivity of ~−0.2 nm/°C, but a sawtooth response of the oscillation wavelength was obtained due to the limited wavelength range for the periodic channeled spectrum of the C-CLPG, which implies the ambiguity in temperature measurement. As result, this property made it necessary to know the initial temperature at the start of the measurement. These properties are in agreement with the results of our previous report [6]. Furthermore, at the end of the tuned wavelength range, dual-wavelength oscillation was obtained instead of single-wavelength, and the temperature-measurement range using single-wavelength oscillation was even narrower than the tuned range in Figure 3, which was desired for practical temperature measurement. However, since the oscillation in different wavelength ranges could be obtained by controlling the VOA loss, a wide single-wavelength oscillation range beyond the limit was considered to be feasible.

**Figure 5:**
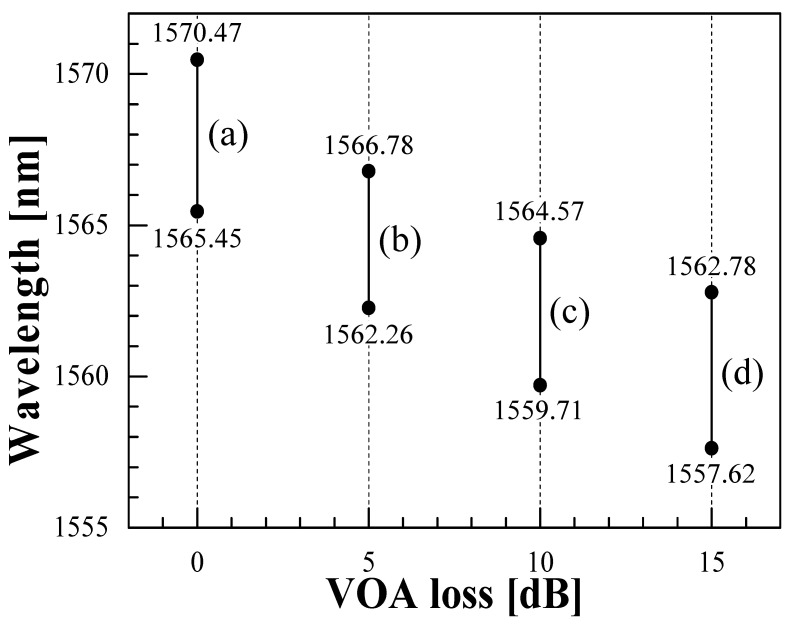
Range of single-wavelength operation for different VOA losses: (**a**) 0 dB, (**b**) 5 dB, (**c**) 10 dB, and (**d**) 15 dB.

As shown in Figure 5, the wavelength ranges of single-wavelength oscillation of the measured spectrum for VOA loss of 0 dB (a), 5 dB (b), 10 dB (c), and 15 dB (d) were 1565.45~1570.47 nm (5.02 nm), 1562.26~1566.78 nm (4.52 nm), 1559.71~1564.57 nm (4.86 nm), and 1557.62~1562.78 nm (5.16 nm); and the total single-wavelength oscillation range was found to be 12.85 nm. When the VOA loss was constant, the oscillation wavelength region, which was continuously shifted without jumping, was limited to the temperature range corresponding to the channel spacing of the C-CLPG. However, by shifting the oscillation wavelength range as shown in Figure 5, the measurement range could be expanded beyond the limitation of temperature change corresponding to the channel spacing of the C-CLPG. Although the expansion of the measurement temperature range by this method was narrower than that by numerical compensation reported previously in [6], a simple technique without the numerical data processing could be provided. From the results obtained, it was possible to measure the change in oscillation wavelength while maintaining single-wavelength operation simply by controlling the VOA loss in three or four steps according to the shift in oscillation wavelength due to temperature change, resulting in a maximum measurable range of the temperature change of ~64 °C. In the present laser configuration, depending on the performance of the VOA used, the tunable range can be further extended toward longer wavelengths by reducing the entire loss of the cavity. Since the C-CLPG used as a sensor element is subjected to disturbances caused by temperature changes and other measurands, the elements other than the sensor part and their connections need to be protected with thermal insulators and cushioning materials or vibration isolators to avoid the unnecessary disturbances, which is a problem for practical applications.

## 4. Concluding Remarks

In this paper, we have proposed an oscillation wavelength range shifting technique based on an intracavity loss modulation in a tunable and switchable EDFRL with a C-CLPG and a VOA to expand the available wavelength range of the previously reported tunable laser configuration [6]. The expansion of the oscillation wavelength range of tunable lasers is an important technology for fiber laser sensors that use an oscillation wavelength as a measurement parameter, from the viewpoint of practical sensor applications. In the laser configuration fabricated, the shifting of the tuning range to the arbitrary wavelength within 1557.62~1570.47 nm was successfully demonstrated by controlling the additional intracavity loss of the VOA, i.e., by expanding the oscillation wavelength range. For the application of the temperature measurement, by controlling the VOA according to the shift of the oscillation wavelength, the single-wavelength oscillation could be maintained over an available temperature range without transitioning to the adjacent channel, which was expected to expand the temperature-measurement range compared to the conventional method. In the experiments, it was shown that single-wavelength oscillation was possible in the range of 1564.57~1565.45 nm (12.85 nm), which corresponded to a temperature range of 64 °C and was equivalent to three times the measurement range realized by the previously reported laser. The proposed laser can be used for two-wavelength oscillation by appropriately controlling the VOA loss, making it useful not only as a sensor, but also as a light source. The improvement of the response time and range for the temperature measurement is an important issue for using this type of sensor in rapid and wide-ranging temperature changes, and is left for further study. In addition, we plan to conduct demonstrations of other parameters that C-CLPG can measure, including refractive index, mechanical deformation, and vibration of the surrounding environment.

## Data Availability

The data presented in this study are available on request from the corresponding author.

## References

[1] Othonos A., Kalli K. (1999). Fiber Bragg Gratings ~Fundamentals and Applications in Telecommunications and Sensing.

[2] Vengsarkar A.M., Lemaire P.J., Judkins J.B., Bhatia V., Erdogan T., Sipe J.E. (1996). Long-period fiber gratings as band-rejection filters. J. Lightwave. Technol..

[3] James S.W., Tatam R.P. (2003). Optical fibre long-period grating sensors: Characteristics and application. Meas. Sci. Technol..

[4] Tanaka S., Tsukida O., Ngo T.T., Wada A., Takahashi N. Simultaneous multipoint strain measurement using cascaded long period fiber gratings. Proceedings of the 24th Optical Fiber Sensors (OFS-24).

[5] Nagatsuka M., Koizumi M., Tanaka S., Wada A., Takahashi N. Precise measurement technique of long period fiber grating sensors using Fourier transform method. Proceedings of the 25th Optical Fiber Sensors (OFS-25).

[6] Fukushima K., Bui Q.H., Nakaya K., Soares M.G., Wada A., Tanaka S., Ito F. (2020). EDF ring laser using cascaded-chirped long period fiber grating for temperature measurement. Opt. Express.

[7] Xie W., Zhang Y., Wang P., Wang J. (2018). Optical Fiber Sensors Based on Fiber Ring Laser Demodulation Technology. Sensors.

[8] Frazão O., Correia C., Baptista J.M., Marques M.B., Santos J.L. (2008). Ring fibre laser with interferometer based in long period for sensing applications. Opt. Commun..

[9] Yan M., Luo S., Zhan L., Zhang Z., Xia Y. (2007). Triple-wavelength switchable Erbium-doped fiber laser with cascaded asymmetric exposure long-period fiber gratings. Opt. Express.

[10] Liu X., Zhan L., Luo S., Wang Y., Shen Q. (2011). Individually Switchable and Widely Tunable Multiwavelength Erbium-Doped Fiber Laser Based on Cascaded Mismatching Long-Period Fiber Gratings. J. Lightwave Technol..

[11] Franco P., Midrio M., Tozzato A., Romagnoli M., Fontana F. (1994). Characterization and optimization criteria for filterless erbium-doped fiber lasers. J. Opt. Soc. Am. B.

[12] Melo M., Frazão O., Teixeira A.L.J., Gomes L.A., Ferreira da Rocha J.R., Salgado H.M. (2003). Tunable L-band erbium-doped fibre ring laser by means of induced cavity loss using a fibre taper. Appl. Phys. B.

[13] Venkitesh D., Vijaya R. A tunable erbium doped fiber ring laser without the use of intra-cavity filters. Proceedings of the Photonics North 2007.

[14] Liu Y., Dong X., Shum P., Yuan S., Kai G., Dong X. (2006). Stable room-temperature multi-wavelength lasing realization in ordinary erbium-doped fiber loop lasers. Opt. Express.

[15] Li F., Feng X., Lu C., Tam H.Y., Wai P.K.A. Characterization of multiwavelength erbium doped fiber lasers with intensity-dependent loss. Proceedings of the 16th Opto-Electronics and Communications Conference (OECC2011).

[16] Guzman-Chavez A.D., Vargas-Rodriguez E., Martinez-Jimenez L., Vargas-Rodriguez B.L. (2021). Switchable multi-wavelength and tunable waveband fiber laser based on a thermal sensitive filter. Opt. Commun..

[17] Fukushima K., Soares M.G., Nakaya K., Wada A., Tanaka S., Ito F. Wavelength Tunable and Switchable EDF Ring Laser using Cascaded-Chirped Long Period Fiber Grating. Proceedings of the 25th Opto-Electronics and Communications Conference (OECC2020).

[18] Bellemare A. (2003). Continuous-wave silica-based erbium-doped fibre lasers. Prog. Quantum Electron..

[19] Saleh B.E.A., Teich M.C. (2007). Theory of laser oscillation. Fundamentals of Photonics.

